# Anti-Inflammatory Effect of Medicinal Fungus *Antrodia cinnamomea* Cultivated on *Pinus morrisonicola* Hayata

**DOI:** 10.17113/ftb.62.03.24.8257

**Published:** 2024-09

**Authors:** Chien-Wei Hou, Bo-Yun Zhao, Shih-Lun Liu, Yuh-Shuen Chen

**Affiliations:** 1 Department of Biotechnology and Pharmaceutical Technology, Yuanpei University of Medical Technology, N0. 306, Yuanpei Street, Hsinchu City, Taiwan; 2 Department of Food Science and Technology, Hungkuang University, No. 1018, Section 6, Taiwan Boulevard, Shalu District, Taichung City, Taiwan; 3 Department of Nutrition, China Medical University, No. 100, Section 1, Jingmao Road, Beitun District, Taichung City, Taiwan; 4 Department of Food Science and Biotechnology, National Chung Hsing University, 145 Xingda Road, South District, Taichung City, Taiwan; 5 Department of Food Nutrition and Health Biotechnology, Asia University, 500 Lioufeng Road, Wufeng District, Taichung City, Taiwan

**Keywords:** *Antrodia cinnamomea*, triterpenoids, antcins, *Cinnamomum kanehirae*, *Pinus morrisonicola* Hayata, anti-inflammation

## Abstract

**Research background:**

The fungus *Antrodia cinnamomea*, which grows on *Cinnamomum kanehirae* tree, has many medicinal uses. However, its cultivation using the traditional method of growing on the *C. kanehirae* tree is costly and time-consuming. A possible alternative method of cultivating *A. cinnamomea* is to use *Pinus morrisonicola* Hayata tree, as it contains α-terpineol, which stimulates the synthesis of triterpenoids.

**Experimental approach:**

To compare the cultivation of *A. cinnamomea* on *P. morrisonicola* and *C. kanehirae*, the contents of triterpenoids and antcin were determined using high-performance liquid chromatography. Anti-inflammatory effects of the extracts of each product were investigated in lipopolysaccharide (LPS)-stimulated BV-2 cells. Their mechanisms on mitogen-activated protein kinase (MAPK) signalling pathways (p38, c-Jun N-terminal kinase (JNK) and extracellular signal-regulated kinase (ERK)) were determined using Western blot analysis.

**Results and conclusions:**

The results showed that the cultivation times of *A. cinnamomea* on *P. morrisonicola* and traditional *C. kanehirae* discs were drastically different, lasting 6 and 18 months, respectively. The concentration of triterpenoids in the corresponding fruiting bodies was (70.0±3.0) and (20.0±4.0) mg/mL, respectively. More antcins were produced in the *P. morrisonicola* culture. Similar anti-inflammatory effect was obtained by both cultures, which is confirmed by the reduced production of IL-1β, IL-6, COX-2 and nitrogen monoxide. Their mechanisms were confirmed by the suppression of MAPK signalling pathways.

**Novelty and scientific contribution:**

Cultivation on *P. morrisonicola* is an innovative and more cost-effective method for growing *A. cinnamomea*. The same anti-inflammatory effect is achieved in a shorter production time.

## INTRODUCTION

*Antrodia cinnamomea* is an indigenous fungus that grows naturally only in the inner cavity of the evergreen tree *Cinnamomum kanehirae* Hayata (Lauraceae). The fruiting body of *A. cinnamomea* is used in folk medicine for its antipyretic, analgesic and hangover relieving effects and for the treatment of liver disease (*1*, *2*). The fungus has been studied for its biological activities, including anticancer and anti-inflammatory properties, liver protection and immunoregulation (*3*-*10*). The main components of *A. cinnamomea* have been identified as polysaccharides, terpenoids, benzenoids and nucleic acids (*11*, *12*). Triterpenoids are crucial compounds found in the fungus and include a diverse range of tetracyclic structures such as ergostanes and lanostanes (*13*). Notably, over 30 steroid-like compounds, mainly antcins, characterised by ergostane skeletons unique to *A. cinnamomea*, constitute approx. 60 % of its dry mass (*14*).

The principle of evaluating the anti-inflammatory properties of foods is based on measuring the effect of food components on changes in the level of inflammatory biomarkers such as cytokines, nitrogen oxide, enzyme activity and other inflammation-related molecules (*15*, *16*). Dietary inflammatory index (*15*), cell-based assays (*16*), co-culture models (*17*), cytokine assays (*18*), NO assay (*16*) and Western blotting (*19*) are the common methods for assessing the anti-inflammatory effects.

However, the fruiting body of *A. cinnamomea* grown on *C. kanehirae* Hayata is in danger of extinction and it usually requires 1–2 years to grow on a cut log by artificial cultivation, but more than 5 years on wild *C. kanehirae* logs (*20*). In addition, the rarity of wild *C. kanehirae* has led to timber theft, causing ecological, environmental and crime problems (*1*). The fruiting body of wild *A. cinnamomea* growing on wild *C. kanehirae* logs is highly valued for its strong medicinal properties (*20*). However, it is extremely rare and difficult to harvest sustainably (*4*). In contrast, the fungus cultivated on a log offers better control, shorter growth time and significantly higher safety (*21*), although the efficiency of artificially cultivated fruiting bodies on *C. kanehirae* logs is inferior to that of wild fruiting bodies (*22*). Extensive research has shown that the efficiency of cultivated *Antrodia cinnamomea* is increasingly comparable to that of wild *A. cinnamomea* (*23*). Moreover, safety assessments of cultivated *A. cinnamomea* show no significant adverse effects, supporting its potential as a safe alternative for human consumption (*24*). Therefore, to meet the market demand, improvement or replacement of *C. kanehirae* culture would be a good strategy to reduce the cost and time of cultivating *A. cinnamomea*.

Recently, α-terpineol has been reported to promote the production of triterpenoids from *A. cinnamomea* mycelia in both submerged and solid-state culture (*25*-*28*). *Pinus morrisonicola* Hayata, known as ’five-leaf pine’, is a native species widely distributed in the mountains of Taiwan. In Taiwan, the pine needle is considered a folk medicine and functional food (*29*). Pine bark and needles contain volatile compounds such as α-terpineol (*30*). In addition, current regulations prohibit the cutting of *C. kanehirae* trees from the natural environment for use in artificial solid-state cultivation. Therefore, using *P. morrisonicola* discs in the production of *A. cinnamomea* could have advantages in terms of availability, cost and environmental protection. To our knowledge, we were the first to use *P. morrisonicola* discs with an innovative compression technique for the cultivation of *A. cinnamomea*.

In the present study, *P. morrisonicola* and *C. kanehirae* disc cultures were explored for the cultivation of *A. cinnamomea* and the content of triterpenoids in these fruiting bodies was investigated. The anti-inflammatory activities of these extracts were evaluated by five different methods and their mechanisms were investigated on the activated extracellular signal-regulated kinase (ERK), c-Jun N-terminal kinase (JNK) and p38 mitogen-activated protein kinase (MAPK) signalling pathways. These results would provide data to support the use of *P. morrisonicola* in the cultivation of this important medicinal fungus, with the expectation that *P. morrisonicola* could replace the traditional use of *C. kanehirae* for the cultivation of *A. cinnamomea* in the future.

## MATERIALS AND METHODS

Antcin standards (antcin A, B, C, H and K), extracted from *Antrodia cinnamomea* and purified, were purchased from Honest & Humble Biotechnology Company (Taoyuan, Taiwan). Reagents were purchased from different sources: lipopolysaccharide, dehydrosulphurenic acid and ursolic acid (Sigma-Aldrich, Merck, St. Louis, MO, USA), antibodies anti-p38 MAPK phospho (pT180/pY182) (Rabbit 1:5000; Cell Signaling Technology, Danvers, MA, USA), anti-SAPK/JNK (Thr183/Tyr185) (Rabbit 1:1000; Calbiochem, Darmstadt, Germany), anti-phospho-p44/42 MAPK (Erk1/2) (thr202/tyr204) (Rabbit 1:1000; Cell Signaling Technology) and anti-beta actin polyclonal antibody (Rabbit 1:1000; Bioss Antibodies, Woburn, MA, USA).

### Strain and cultural condition

*Antrodia camphorata* was collected in Taiwan. The species was identified by Testing and Analysis Center for Food and Cosmetics (ISO 17025) of Hungkuang University, Taichung, Taiwan. The strain was kindly provided by Holy Stone Biotechnology Company (Taichung, Taiwan). The strain of *A. camphorata* was preserved in potato dextrose agar (PDA) slants and stored at 4 °C. The plate with PDA medium was used for cultivation of mycelia transferred from the stock slants at 26 °C for 21 days. A volume of 10 mL of 0.9 % NaCl solution was used to wash arthroconidia. The spore count of washed arthroconidia needs to be adjusted to be observed by haemocytometry (*27*). For submerged culture, the spore count of adjusted arthroconidia was 10^7^/mL. A volume of 100 mL of medium (3.3 g glucose, 0.28 g peptone, 0.2 g soybean flour, 0.2 g wheat bran extract, 0.2 g KH_2_PO_4_ and 1.0 g MgSO_4_) was filled in the 500-mL Erlenmeyer flask and incubated at 26 °C and shook at 10.5 rad/s for 12 days. Before incubation, the pH of the culture medium was adjusted to 4.5.

### Solid culture preparation

The round *C. kanehirae* wood block was cut into (20±2) cm in diameter and (10±1) cm in height and a hole was drilled into the centre of the block ((2±0.1) cm in diameter and (0.5±0.1) cm in depth). After the sterilisation of the wood, 3 mL of arthroconidia were implanted in the central hole. The wood was placed in a sealed sterile bag and incubated without light at 26 °C under a controlled atmosphere (carbon dioxide 4000 mg/L).

Shredded branches and leaves of *P. morrisonicola* were extruded into a round block ((20±2) cm in diameter and (10±1) cm in height) with a hole drilled into the centre ((2±0.1) cm in diameter and (0.5±0.1) cm in depth). The sterilisation and incubation of the *P. morrisonicola* wood block was the same as that of the *C. kanehirae* wood block. Three *C. kanehirae* and three *P. morrisonicola* wood blocks were incubated at the same time in this study.

### Quantification of triterpenoids

The vanillin-acetic acid system was used for quantification of triterpenoids (*31*). Freeze-dried fruiting body (1 g) of *A. cinnamomea* was extracted by ultrasonic vibration (DC80; DELTA, Tainan, Taiwan) with 10 mL methanol for 30 min at room temperature. The fruiting body precipitate was removed by centrifugation at 3100×*g* and 4 °C for 10 min (CR21G; HITACHI, Tokyo, Japan) and the supernatants were collected for the assay. A volume of 0.2 mL supernatant was mixed with 0.4 mL of 5 % (*m*/*V*) vanillin-acetic acid solution and 1.6 mL perchloric acid. Then 4.0 mL ethyl acetate were added and the absorbance was measured at 560 nm (U2000; HITACHI, Tokyo, Japan). The standard curve for determination of triterpenoids was built up by ursolic acid (0.2 g/L) (*31*).

### Quantification of antcins

Freeze-dried fruiting body (1 g) of *A. cinnamomea* was extracted by ultrasonic vibration with 10 mL methanol for 30 min at room temperature. The centrifugation (CR21G; HITACHI) at 3100×*g* was used to remove the remaining fruiting body. The triterpenoids were analysed using high-performance liquid chromatography (HPLC; Agilent Technologies, Santa Clara, CA, USA) with filtrate filtered through 0.22-μm membrane. The C18 reversed-phase column (4.6 mm×250 mm, 5 μm; Waters XBridge, Framingham, MA, USA) was used for analysis. The mobile phase consisted of solvent A (ultrapure water containing 0.1 % formic acid) and solvent B (acetonitrile). The flow rate was 1.0 mL/min. The gradient conditions were set at 55-15 % A for 0–12 min, 45-40 % A for 12–17 min, 40-0 % A for 17–26 min, and 0 % A for 26–40 min. Column temperature was set at 35 °C. Peaks were detected using UV detector (*λ*=254 nm) or diode array detector (DAD) (*λ*=190–400 nm). Injection volume was 10 μL (*32*). The antcin A, B, C, H, K and dehydroeburicoic acid were dissolved in methanol, their peaks were identified using individual standards and their retention times were confirmed. The correlation coefficients (r) of standard curves of antcin A, B, C, H, K and dehydroeburicoic acid were 0.9903, 0.9847, 0.9896, 0.9953, 0.9921 and 0.9862, respectively.

### Cell culture

The humidified incubator containing 5 % CO_2_ was used for preservation of murine microglial BV-2 cells with Dulbecco's modified Eagle's medium (DMEM, Gibco, Grand Island, NY, USA) at 37 °C. The modified medium was supplemented with 10 % foetal bovine serum (FBS, 5 % heat-inactivated horse serum), 100 U/mL penicillin and 100 μg/mL streptomycin. Confluent cultures were passaged by trypsinisation (*33*, *34*). BV-2 cells, washed twice with warm serum-free DMEM, were reacted with *A. cinnamomea* extract and lipopolysaccharide (LPS) for the indicated times. The *A. cinnamomea* extracts were diluted with 95 % ethanol (*V*(extract):*V*(ethanol)=1:10).

### Cell viability

The metabolised blue formazan with 3-(4,5-dimethyl-thiazol-2-yl)-2,5-diphenyl tetrazolium bromide (MTT) was used for measuring cell viability as the mitochondrial dehydrogenase in living cells. BV-2 cells were incubated at 5·10^5^ cells per well in 24-well plates for 24 h and washed with PBS (*35*). The cells were then treated with *A. cinnamomea* extracts at various concentrations and incubated at 37 °C for 24 h. Following this, the cells were reacted with 0.5 mg/mL MTT reagent for 1 h to assess cell viability. After reaction for 1 h, 200 μL of solubilisation solution were added. The absorbance was measured at 540 nm with microplate reader (SpectraMAX 340; Molecular Devices, Sunnyvale, CA, USA). The assay was performed according to manufacturer’s procedures (Thermo Fisher Scientific, Waltham, MA, USA). The results were presented as the mean percentage of viable cells *vs* the control (BV-2 cells were treated identically to the experimental group but without *A. cinnamomea* extracts).

### Lactate dehydrogenase release assay

The supernatant of BV-2 cells was collected to determine the cytotoxicity by the release of lactate dehydrogenase (LDH). LDH is a cytosolic enzyme that is released into the culture media when the plasma membrane is permeable and serves as an indicator of cell membrane integrity. The 3 μg/mL LPS were added to *A. cinnamomea* extracts and reacted for 24 h. The potassium phosphate buffer with nicotinamide adenine dinucleotide (NADH) and 0.2 mL sodium pyruvate were mixed and adjusted to 0.1 mL of cell-free supernatant. The supernatant was then injected to a 96-well plate and the absorbance was recorded at 490 and 630 nm with microplate reader (Multiskan SKY microplate spectrophotometer; Thermo Fisher Scientific, Taipei City, Taiwan) (*34*). The results were presented as the mean percentage of viable cells *vs* the LPS control.

### Cytokine assay

BV-2 cells at various cell counts, with a maximum at 10^5^ cell/mL in a 96-well plate, were treated with 3 μg/mL LPS for 24 h in the presence of *A. cinnamomea* extracts. The cytokine and cyclooxygenase-2 (COX-2) production were determined in the supernatant. Concentrations of interleukins IL-1β (pg/mL) and IL-6 (pg/mL), and COX-2 (ng/mL), were determined with Mouse IL-1 beta SimpleStep ELISA® kit, Murine IL-6 Mini ABTS ELISA Development kit and COX2 SimpleStep ELISA® kit (Abcam, Boston, USA), respectively (*34*).

### Nitrogen monoxide assay

Nitrogen monoxide (NO) from the culture supernatant was measured as nitrite. BV-2 cells were treated with LPS alone or combined with *A. cinnamomea* extracts from the fruiting bodies grown on *C. kanehirae* or *P. morrisonicola* discs for 24 h. Nitrite concentration (μM) in the supernatants collected from treated and non-treated cell cultures was determined using Griess test and the absorbance was measured at 540 nm with a microplate reader (*35*).

### Preparation of cell extracts

The BV-2 cells were reacted with *A. cinnamomea* extracts (with and without 3 μg/mL LPS) for 30 min, collected and centrifuged at 3100×*g* (CR21G; HITACHI). The appropriate resuspension volume of the lysis buffer was about 4·10^7^ cell/mL for cell pellets. The lysis buffer consisted of 10× RIPA lysis buffer (Upstate, Lake Placid, NY, USA), 137 mM NaCl, 20 mM Tris-HCl, 1 mM phenylmethylsulfonylfluoride, 10 μg/mL leupeptin, 10 μg/mL aprotinin and 5 μg/mL pepstain. The pH of the mixed buffer was adjusted to 7.5. The suspension of cells was then sonicated. The Bradford assay (Bio-Rad, Hemel Hempstead, Hertfordshire, UK) was used for determination of protein concentration. Protein was extracted by RIPA lysis buffer and adjusted to the concentration of 25 μg/μL, then heated at 95 °C for 10 min for the Western blotting experiment. The results are shown as a relative ratio of proteins (% of LPS control). The 2 mg/mL lysis buffer was used for the adjustment of the cell lysates (*34*).

### Western blotting

The 12 % sodium dodecyl sulfate-polyacrylamide gel electrophoresis was used to separate the protein extracts. Proteins were transferred into polyvinylidene difluoride membranes (Millipore, Bedford, MA, USA) and blocked with 5 % dry skimmed milk in Tris-buffered saline with 0.1 % Tween® 20 (TBST) for 2 h at room temperature. Then the membranes were incubated with antibodies including anti-COX-2 of rabbit (1:1000; Cayman Chemical, Ann Arbor, MI, USA) and anti-phospho-MAPKs, followed by incubation with secondary antibody: streptavidin-horseradish peroxidase conjugated goat anti-rabbit IgG antibody (Jackson ImmunoResearch, West Grove, PA, USA) (*33*, *34*).

### Statistical analysis

Data of *in vitro* experiments were presented as mean value±standard deviation (S.D.). The Student's *t*-test was used for single variable comparisons. For multiple variable comparisons, one-way analysis of variance (ANOVA) with Scheffe's test was used. The data were considered significant with p-values less than 0.05 (*34*).

## RESULTS AND DISCUSSION

### Content of triterpenoids

The fruiting bodies of *Antrodia cinnamomea* (*d*>0.5 cm) were harvested and the cultivation times on the discs of *Pinus morrisonicola* and *Cinnamonum kanehirae* were 6 and 18 months, respectively. Total concentrations of triterpenoids from the dried fruiting bodies of *A. cinnamomea* were (70.0±3.0) and (20.0±4.0) g/mL, obtained on the discs of *P. morrisonicola* and *C. kanehirae*, respectively. The mass of cultivated fruiting bodies was (30.2±3.9) and (50.6±4.6) g, respectively, on the *P. morrisonicola* and *C. kanehirae* discs (Table 1).

**Table 1:** Properties of extracts from the fruiting body of *Antrodia cinnamomea* grown on two types of discs

Property	*Cinnamomum kanehirae* disc	*Pinus morrisonicola* Hayata disc (branches and leaves)
*d*(fruiting body)/cm	0.5	0.5
*t*(growth)/month	18	6
*m*(fruiting body)/g	50.6±4.6	30.2±3.9
*m*(extract)/g	2.7±0.4	2.1±0.3
*m*(fruiting body)/g*	18.5	14.3
*γ*(triterpenoids)/(mg/mL)	20.0±4.0	70.0±3.0

*Extraction efficiency expressed as mass of *A. cinnamomea* fruiting body used to obtain 1 g of extract

Our results show that *P. morrisonicola* is a promising replacement for the traditional cultivation of the fungus on discs. The *A. cinnamomea* fruiting body from the culture on *P. morrisonicola* disc produced more triterpenoids than the fruiting body from the culture on *C. kanehirae* disc. In this study, the circulation of carbon dioxide at 4000 mg/L was maintained using a controlled atmosphere technique. The dehydroeburicoic acid concentration of the fruiting body of *A. cinnamomea* on *C. kanehirae* disc after 18 months of cultivation was 2.63 mg/mL, which was significantly higher than that in another report (*36*), where it ranged from 0.63 to 1.02 mg/mL after 22 months of cultivation under normal atmosphere. Although extracts from the fruiting body of *A. cinnamomea* had similar anti-inflammatory activities, the *A. cinnamomea* fruiting body from the culture on the *P. morrisonicola* disc took only a third of culturing time to grow compared with that of the traditional growth on *C. kanehirae* disc (Table 1). Therefore, this new method under controlled atmosphere could improve the production of *A. cinnamomea*, promote its medicinal applications and probably bring economic and ecological benefits.

Because *A. cinnamomea* grown on *C. kanehirae* is rare in nature and usually needs more than a year to grow on a cut log, several methods have been tried to enhance its production (*25*-*28*). Among them, volatile compounds have been used to stimulate the culture of *A. cinnamomea* (*27*, *28*). In a study of submerged culture, α-terpineol (0.5 mg/L) stimulated the triterpenoid content and triterpenoid production by *A. cinnamomea* (*27*, *28*). The triterpenoid content of the fruiting bodies cultivated in this study was higher than the triterpenoid mass fraction of 23.31 mg/g reported in the previous study (*27*). We also chose *P. morrisonicola* for our study because of its content of α-terpineol and other volatile compounds (*30*). Pine needles of *P. morrisonicola* and other pines are known for their volatile compounds and their herbal tea is known to have antioxidant and anti-inflammatory effects (*29*, *37*). Our results showed that *P. morrisonicola* was useful for the cultivation of *A. cinnamomea*. The fruiting body of *A. cinnamomea* from the *P. morrisonicola* disc culture had a shorter production time and more triterpenoids than that from the *C. kanehirae* disc (Table 1).

Recently, the genome for the biosynthesis of sesquiterpenoids, triterpenoids, ergostanes, antroquinonol and antrocamphin in *A. cinnamomea* has been well studied (*38*). Genes were identified that are differentially expressed between the mycelium and the fruiting body. Proteins responsible for the production of medicinal secondary metabolites are also found in several pathways. However, the genes and protein pathways that are affected by α-terpineol for triterpenoid production remain unclear and require further investigation.

### Analysis of antcins

**Fig. 1:**
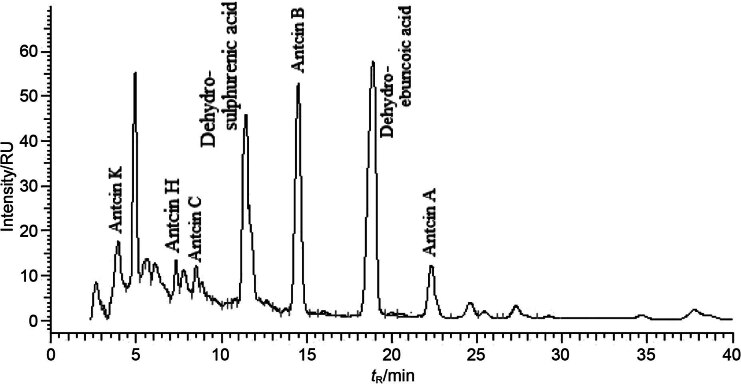
The chromatogram of triterpenoids found in *Antrodia cinnamomea.* Peaks were detected using UV detector at *λ*=254 nm

Antcins A, B, C, H and K, dehydrosulphurenic acid and dehydroeburicoic acid were quantified by HPLC. The chromatogram of the triterpenoids is shown in Fig. 1. The culture grown on *P. morrisonicola* disc produced more antcins in 6 months than the culture grown on *C. kanehirae* disc in 18 months. The total antcin concentration produced on *P. morrisonicola* and *C. kanehirae* disc cultures was 18.10 and 13.19 mg/mL, respectively (Table 2).

**Table 2:** Concentration of triterpenoids in the fruiting body of *Antrodia cinnamomea* grown on two types of discs

	*Cinnamomum kanehirae*				*Pinus morrisonicola* Hayata	
*t*(growth)/month					
3	6	18	3		6
Triterpenoid	*γ*(triterpenoid)/(mg/mL)					
Antcin A	0.59±0.03	1.02±0.03	1.64±0.05	1.08±0.06		2.18±0.07
Antcin B	0.53±0.02	0.84±0.02	2.35±0.09	1.84±0.06		3.9±0.1
Antcin H	N.D.	0.57±0.02	1.42±0.04	0.93±0.03		1.18±0.08
Dehydrosulphurenic acid	0.12±0.01	0.43±0.03	1.68±0.09	1.01±0.03		2.24±0.09
Antcin C	0.61±0.04	0.69±0.03	1.79±0.04	1.31±0.04		2.1±0.1
Antcin K	0.51±0.02	0.78±0.04	1.68±0.08	1.26±0.05		2.35±0.08
Dehydroeburicoic acid	0.58±0.03	0.96±0.04	2.6±0.2	1.99±0.07		4.1±0.1
Total	2.95	5.30	13.19	9.41		18.10

Results are expressed as mean value±standard deviation, *N*=3. N.D.=not detected

Cultivation on *P. morrisonicola* disc yielded significantly more antcins A, B, C, H and K than cultivation on *C. kanehirae* disc (Table 2). The antcin mass fraction in the fruiting bodies cultivated on *C. kanehirae* disc in this study was higher than the 0.5–1.5 mg/g reported by Qiao *et al.* (*13*). Terpenoids, including triterpenoids, meroterpenoids, sesquiterpenoids, diterpenoids and steroids have been well characterised (*11*). Particularly, the ergostane-type triterpenoids (antcins) and the meroterpenoids (antroquinonols) have been characterised as constituents of *A. cinnamomea*. Recent studies have shown that extracts of *A. cinnamomea* and their active components, such as antcins A-K, antrodins A-C, antroquinonols, eburicoic acid and ulphurenic acid, have potent anticancer (*3*, *4*), hepatoprotective (*5*, *6*), anti-inflammatory (*7*-*10*) and antidiabetic (*39*, *40*) properties.

### Anti-inflammatory effect

To test the effect of *A. cinnamomea* extracts on cell viability, BV-2 cells were treated with *A. cinnamomea* extracts for 24 h. First, a pretest for nitrite inhibition was conducted with six different concentrations of *A. cinnamomea* extract: 0.01, 0.1, 1, 10, 50 and 100 μg/mL in cell culture. *A. cinnamomea* extract at concentrations of 0.01 and 0.1 was unable to inhibit nitrite production. At a concentration of 1 μg/mL, the extract of *A. cinnamomea* grown on *P. morrisonicola* inhibited 10 % of nitrite production. However, higher concentrations of *A. cinnamomea* extracts (50 or 100 μg/mL) did not inhibit nitrite better than the concentration of 10 μg/mL. The results showed that the concentrations of 1 to 10 μg/mL had the highest sensitivity in the nitrogen monoxide assay. Therefore, the concentrations of *A. cinnamomea* extract of 1, 5 and 10 μg/mL were selected for subsequent experiments in this study. Lipopolysaccharide (LPS) is an important reagent used in cell-based anti-inflammatory testing because it induces strong inflammatory responses by binding to Toll-like receptor 4 (TLR4) on immune cells, triggering the production of proinflammatory cytokines such as IL-1β, IL-6 and TNF-α (*41*).

When the murine microglial BV-2 cell is stimulated by LPS, ’resting’ form morphologically transforms to an ’activated’ form. The activation of BV-2 cell can in turn cause the release of potentially cytotoxic molecules such as proinflammatory cytokines, reactive oxygen intermediates (ROS), nitrogen monoxide and chemotactic cytokines. The activated BV2 cell has widely expressed intracellular signals that regulate gene expression as a response to the LPS-induced stress. In this study, the direct effects and underlying mechanisms were clarified using the BV2 murine microglia cell line with inflammatory properties (*42*).

**Fig. 2:**
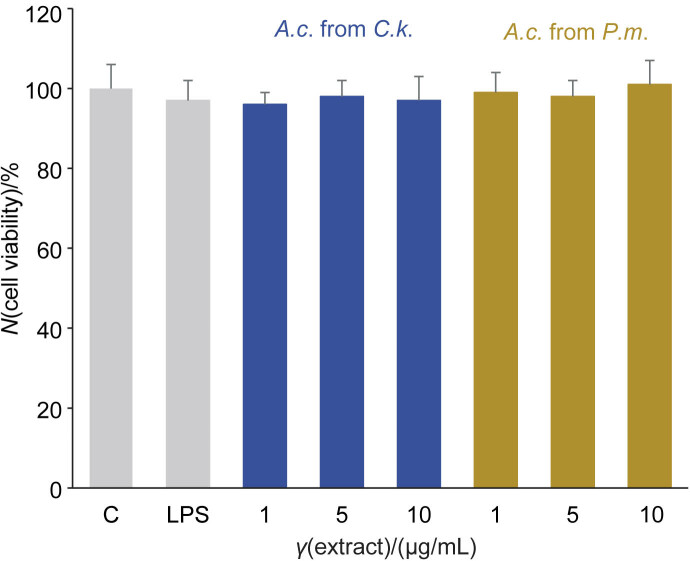
Cell viability test for the *Antrodia cinnamomea* (*A.c*.) extracts. BV-2 cells were treated with *γ*(extract)=1, 5 or 10 μg/mL for 24 h. The MTT results showed no changes in the cell viability. Values represent the mean±S.D., *N*=3. C=untreated control, LPS=lipopolysaccharide-stimulated BV-2 cells, *C.k*.=*Cinnamomum kanehirae*, *P.m*.=*Pinus morrisonicola* Hayata disc

The results of the cell viability test showed that cell viability was not affected by either extract at these concentrations (Fig. 2).

**Fig. 3:**
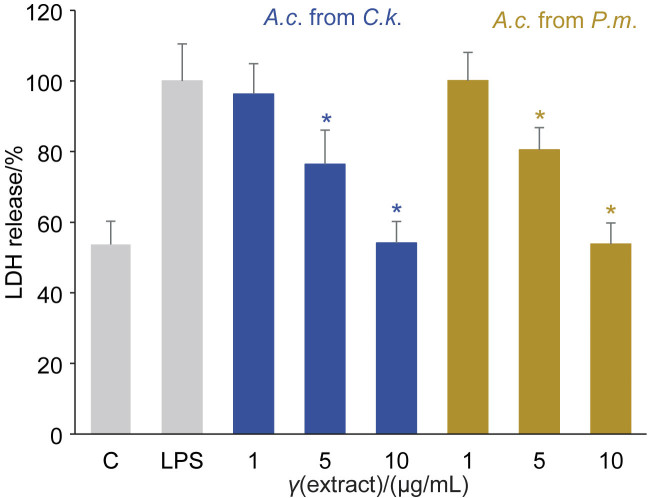
Protection from cell injury. Cells were treated with lipopolysaccharide *γ*(LPS)=3 μg/mL alone and/or in combination with *Antrodia cinnamomea* (*A.c*.) extracts (*γ*=1, 5 or 10 μg/mL) for 24 h. The release of lactate dehydrogenase (LDH) expressed as a percentage of LPS as control was reduced significantly by both extracts. Values represent the mean±S.D., *N*=3. C=untreated control, C.*k.*=*Cinnamomum kanehirae*, *P.m*.=*Pinus morrisonicola* Hayata. *Significant difference between the LPS alone and LPS with *A. cinnamomea* extracts determined using Scheffe's test (p<0.05)

The protective effect of *A. cinnamomea* extracts was investigated in BV-2 cells under LPS-induced injury. Cells were treated simultaneously with *γ*(LPS)=3 μg/mL and *γ*(*A. cinnamomea* extract)=1, 5 or 10 μg/mL for 24 h. The results showed that the release of lactate dehydrogenase (LDH) was reduced by both extracts in a dose-dependent manner (Fig. 3). After 24 h, LPS increased cell toxicity in the LDH assays, nitrite release and the amount of cytoplasmic calcium (*43*). Our experimental results are very similar to a previous report investigating the protective effect of *Hericium erinaceus* mycelium (HEM) extracts in BV-2/N2a cells exposed to LPS-induced injury. The cell viability of the treated cells increased (*42*).

**Fig. 4:**
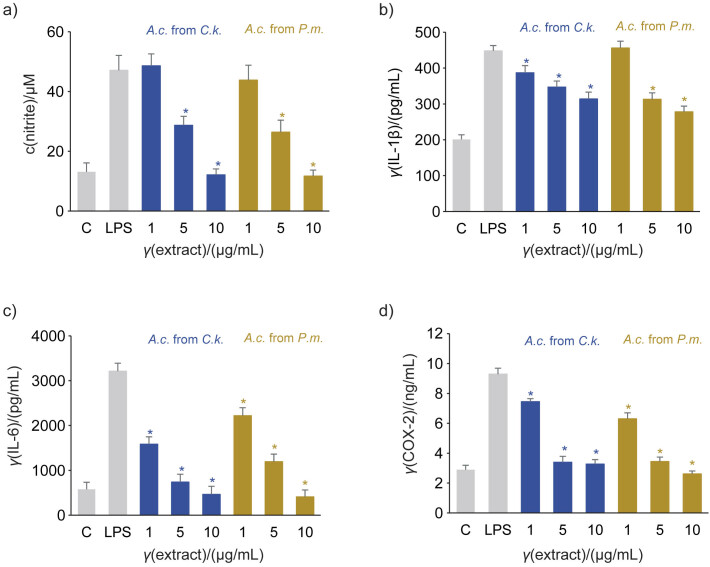
Comparison of anti-inflammatory activity of the two types of *Antrodia cinnamomea (A.c*.) extracts: a) nitrogen monoxide (NO), b) interleukin (IL)-1β, c) IL-6 and d) cyclooxygenase-2 (COX-2) shows the indicated inflammatory mediators. BV-2 cells were treated with 3 μg/mL lipopolysaccharide (LPS) alone or in combination with the extracts of 1, 5 or 10 μg/mL fruiting body of *A. cinnamomea* grown on *Cinnamomum kanehirae* (*C.k.*) or *Pinus morrisonicola* Hayata (*P.m*.) disc for 24 h. Supernatants of the treated and untreated cell cultures were collected and analysed as described in the methods. Results are presented as the mean value±S.D., *N*=3. C=untreated control. *Significant difference between the LPS alone and LPS with *A. cinnamomea* extracts determined using Scheffe's test (p<0.05)

The anti-inflammatory effect of the fungus grown on *P. morrisonicola* and *C. kanehirae* was compared. Supernatants from the cell culture stimulated with LPS and treated with *A. cinnamomea* were collected and analysed. The results showed a dose-dependent anti-inflammatory effect of both extracts. At a concentration of 10 μg/mL, the extract from *A. cinnamomea* fruiting body grown on *P. morrisonicola* disc had a significantly better inhibition of IL-1β ((38±3) *vs*. (30±2) %, respectively) and COX-2 ((71.9±2.1) *vs* (64.8±3.2) %, respectively) than those on traditional *C. kanehirae* disc, but an equal production of NO ((24.7±2.3) *vs* (25.5±2.6) %, respectively) and IL-6 ((87±5) *vs* (86±4) %, respectively) (Fig. 4). The study by Lee *et al.* (*44*) showed that the concentration of IL-6 in *A. cinnamomea* at 50 µM/mL was 400 pg/mL. In contrast, the *A. cinnamomea* cultured in this study showed a superior anti-inflammatory effect.

**Fig. 5:**
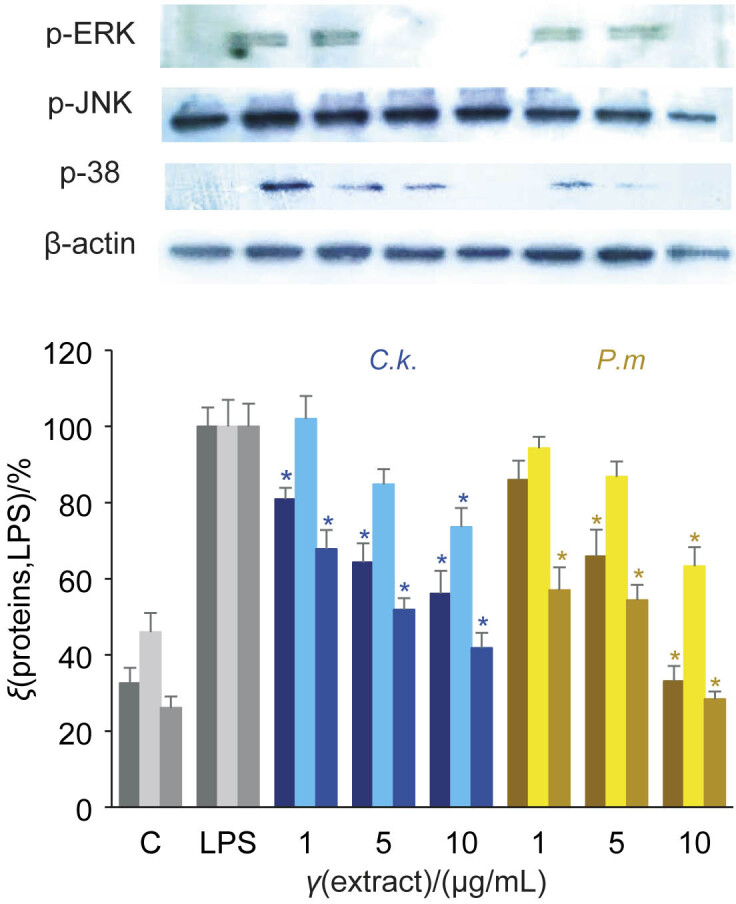
Suppression of mitogen-activated protein kinase (MAPK) signalling pathway. BV-2 cells stimulated with 3 μg/mL lipopolysaccharide (LPS) alone or in combination with the extracts of *Antrodia cinnamomea γ*=1, 5 or 10 μg/mL for 30 min. Cells were extracted and analysed by Western blotting. The results showed that the extracts of the fruiting body of *A. cinnamomea* grown on *Cinnamomum kanehirae* (*C.k*.) or *Pinus morrisonicola* (*P.m*.) disc had a similar pattern of suppression of p38, JNK and ERK/MAPK signalling pathways. Results represent the mean value±S.D., *N*=3. C=untreated control. *Significant difference between the LPS alone and LPS with the extracts of *A. cinnamomea* determined using Scheffe's test (p<0.05)

Fig. 5 shows the suppression of the mitogen-activated protein kinase (MAPK) signalling pathway. BV-2 cells were stimulated by LPS alone or in combination with the extracts of *A. cinnamomea* for 30 min. BV-2 cells stimulated with 3 μg/mL LPS and treated with 10 μg/mL *A. cinnamomea* were extracted and analysed by Western blotting. MAPKs are thought to be involved in the regulation of cell survival and cell death under numerous conditions (*45*). In many cell types, numerous extracellular stimuli regulate growth, differentiation and apoptosis through the activation of protein kinase cascades. LPS can activate several signalling pathways of MAPKs: the extracellular signal-regulated protein kinase (ERK1/2), the c-Jun N-terminal kinase (JNK) and the p38 MAPK. We therefore investigated whether the protective effect of *A. cinnamomea* extracts is mediated by the inhibition of MAPK and apoptosis pathways in BV-2 cells stimulated with LPS.

Our results show that *A. cinnamomea* extracts grown on both discs of *C. kanehirae* and *P. morrisonicola* similarly suppressed MAPK signalling pathways: p38, JNK and ERK at (71.6±2.0), (36.6±5.0) and (66.9±4.0) % *vs* (58.2±4.0), (26.4±5.0) and (43.9±6.0) %, respectively (Fig. 5).

The results also show that the anti-inflammatory effect of the two *A. cinnamomea* extracts was similar. The extract from the *P. morrisonicola* disc had slightly better results than that from *C. kanehirae* in inhibiting IL-1β and COX-2, but acted equally on NO and IL-6 production. The suppression of LPS-induced p38, JNK and ERK/MAPK signalling pathways was consistent with the anti-inflammatory effects of *A. cinnamomea* in the previous reports (*46*). In addition, the water extract of *A. cinnamomea* also suppresses the LPS-stimulated NF-κB signalling pathway in macrophages and leads to down-regulated NO production, inducible nitrogen monoxide synthase (iNOS) and COX-2 protein levels, and inflammatory cytokines (*47*). Interestingly, a recent finding shows that antcin K from *A. cinnamomea* inhibits important mediators of rheumatoid arthritis such as tumour necrosis factor alpha (TNF-α), IL-8 and IL-1β in human rheumatoid synovial fibroblasts. The mechanism involves the suppression of the phosphorylation of the phosphoinositide 3-kinase, focal adhesion kinase, protein kinase B and NF-κB signalling pathways. The intraperitoneal injections of antcin antroquinonols (10 or 30 mg/kg) into ankle joint tissue samples from a collagen-induced arthritis (CIA) mouse model showed attenuation of cartilage degradation, bone erosion and paw swelling. The TNF-α, IL-1β, IL-6 and IL-8 were also reduced in this model (*48*). A recent study of human Phase I in healthy volunteers showed that *A. cinnamomea* extract LEAC-102 was safe and well-tolerated in healthy adults. The potential immunomodulatory function of *A. cinnamomea* extract was observed (*49*). These results could further expand the clinical use of *A. cinnamomea* to treat many diseases.

The recent COVID-19 pandemic, caused by the novel coronavirus SARS-CoV-2, has taken a worldwide toll on health, economy and human lives. Importantly, SARS-CoV-2 enters host cells through the receptor angiotensin-converting enzyme 2 (ACE2) (*50*). ACE2 is both an enzyme and a functional receptor on the cell surface that is highly expressed in the lungs, heart and kidneys and is secreted into the blood. A recent report shows that treatment with various antcins (A, B, C, H and K) can significantly inhibit ACE2 activity in human epithelial cells, suggesting a potential application to combat COVID-19 infection (*51*). In addition, a recent study has shown that antcin A can regulate the metabolic changes induced by the SARS-CoV-2 spike protein in phorbol 12-myristate 13-acetate-induced human monocyte THP-1 cells (*52*). Since the fruiting body of *A. cinnamomea* grown on *P. morrisonicola* produced more triterpenoids (including antcins A, B, C, H and K) than that grown on *C. kanehirae*, it may facilitate future research and applications.

## CONCLUSIONS

In conclusion, we have found that cultivation of the fungus *Antrodia cinnamomea* on the log of *Pinus morrisonicola* is a better alternative to the traditional cultivation on *Cinnamonum kanehirae*. Although both methods showed the same anti-inflammatory effect, the *P. morrisonicola* disc requires a shorter cultivation time. This would be a great advantage in terms of production costs, environmental protection and the wider use of *A. cinnamomea* in healthcare.
